# The prognostic effect of residual tumor for advanced epithelial ovarian cancer treated with neoadjuvant chemotherapy or primary debulking surgery

**DOI:** 10.1002/cam4.4642

**Published:** 2022-03-10

**Authors:** Shi‐Ping Yang, Jian‐Xian Chen, Jing‐Ying Xu, Jian Lei, San‐Gang Wu, Juan Zhou

**Affiliations:** ^1^ Department of Radiation Oncology Hainan General Hospital (Hainan Affiliated Hospital of Hainan Medical University) Haikou People's Republic of China; ^2^ Department of Medical Oncology People's Hospital of Baise Baise People's Republic of China; ^3^ Department of Obstetrics and Gynecology, the First Affiliated Hospital of Xiamen University School of Medicine, Xiamen University Xiamen People's Republic of China; ^4^ Department of Radiation Oncology, the First Affiliated Hospital of Xiamen University School of Medicine, Xiamen University Xiamen People's Republic of China

**Keywords:** debulking surgery, epithelial ovarian cancer, neoadjuvant chemotherapy, residual tumor, survival

## Abstract

**Purpose:**

The role of neoadjuvant chemotherapy (NACT) and primary debulking surgery (PDS) in advanced epithelial ovarian cancer (EOC) remains controversial. This study aimed to investigate the prognosis between NACT and PDS in advanced EOC. We also investigated the prognostic effect of the residual tumor (RT) after NACT and PDS.

**Methods:**

Patients with stage III‐IV EOC diagnosed between 2010 and 2017 were included from the Surveillance, Epidemiology, and End Results (SEER) database. Chi‐square test, multivariate logistic regression analysis, Kaplan–Meier curves, and Cox proportional hazards model were used for statistical analyses.

**Results:**

A total of 5522 women patients were identified, 2017 (36.5%) and 3505 (63.5%) patients received NACT and PDS, respectively. There were 2971 (53.8%), 1637 (29.6%), and 914 (16.6%) patients who had no residual tumor, RT ≤1 cm, and RT >1 cm, respectively. There were 25.5% of patients receiving NACT in 2010 and 48.4% in 2017 (*p* < 0.001). Women treated with NACT were not related to a higher chance of complete resection than the PDS group (*p* = 0.098). Patients receiving PDS had significantly better cancer‐specific survival (CSS) than those receiving NACT (*p* < 0.001). The 5‐year CSS was 35.3% and 51.1% in those receiving NACT and PDS, respectively. In patients receiving NACT, those who had no residual tumor had significantly better CSS compared to those who had RT ≤1 cm (*p* < 0.001), while comparable CSS was found between those who had RT ≤1 cm and RT >1 cm (*p* = 0.442). In those receiving PDS, the CSS was decreased with a RT increase (*p* < 0.001).

**Conclusions:**

Our study suggests that PDS may be the optimal procedure for the majority of advanced EOC patients. Complete resection of all residual diseases should be the goal with the increased utilization of NACT.

## Graphical abstract

Our study aimed to investigate the prognosis between neoadjuvant chemotherapy and primary debulking surgery in advanced epithelial ovarian cancer. Our study suggests that primary debulking surgery may be the optimal procedure for the majority of patients with advanced epithelial ovarian cancer. Complete resection of all residual diseases should be the goal with the increased utilization of neoadjuvant chemotherapy.

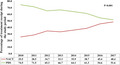

## 1 INTRODUCTION

Epithelial ovarian cancer (EOC) is the lead cause of death for major gynecologic malignancies, with approximately 70% of patients having an advanced‐stage disease (stage III–IV) and an inferior overall survival (OS) was found in this patient subset.^1^ The standard management in advanced EOC is primary debulking surgery (PDS), followed by taxane and platinum adjuvant chemotherapy according to several society guidelines.^2^, ^3^ Neoadjuvant chemotherapy (NACT) and delayed interval debulking surgery (IDS) are alternative options based on the perspective and retrospective studies, which showed a similar prognosis compared to PDS as well as increased optimal debulking rate, improved quality of life, and decreased surgery‐related complications.^4^, ^5^, ^6^, ^7^, ^8^ In the current clinical practice, NACT followed by IDS is increasingly used in advanced EOC.^9^ However, although there are still controversies, the utility of NACT followed by IDS is increasing in advanced EOC.

The European Organization for Research and Treatment of Cancer (EORTC) 55,971 and Chemotherapy OR Upfront Surgery (CHORUS) randomized trials reported similar prognoses between NACT and PDS.^4^, ^5^ However, the major limitation of these trials is a low number of patients who had tumor completely resection (18%–20.3% in the PDS group and 29%–52.1% in the NACT group). Moreover, the median survival time was significantly shorter than other single‐institution studies.^10^, ^11^ Regarding the utility of NACT in advanced EOC, conflict results were found in a survey between the European Society for Gynecological Oncology (ESGO) and the United States (US) Society of Gynecologic Oncology (SGO). In SCO, 82% of members believed that the existing evidence was insufficient to support the utility of NACT,^12^ while 70% of the ESGO members believed that there was enough evidence to support the use of NACT for advanced EOC.^13^ Several previous studies also raised concerns about increased resistance to platinum among patients receiving NACT.^14^, ^15^ In light of this, our study aimed to assess the survival outcomes between NACT and PDS in stage IIIC/IV EOC. More specifically, we also investigated the prognostic effect of the residual tumor (RT) after NACT and PDS, which would add to the current knowledge of treatment decision‐making in advanced EOC.

## 2 MATERIALS AND METHODS

### 2.1 Patients

We extracted EOC data from the Surveillance, Epidemiology, and End Results (SEER) program between 2010 and 2017. The SEER database covers approximately 47.9% of the US population, which includes de‐identified information regarding cancer incidence, demographics, clinicopathological characteristics, treatment as well as survival status. We identified patients who met the following criterion: (1) diagnosed with stage IIIC–IV high‐grade serous ovarian cancer, (2) received NACT and IDS or PDS + adjuvant chemotherapy, (3) available data for age, race, RT status, and serum levels of CA125 before treatment. Patients who received intraoperative systemic therapy or received surgery both before and after systemic therapy were excluded. This study did not require Institutional Review Board approval due to the de‐identified patient information in the SEER dataset.

### 2.2 Variables

We included the following variables in this study: age, year of diagnosis, race, tumor stage, RT status, the levels of CA125 before treatment, and the receipt of NACT or PDS. The tumor stage was based on the 7th American Joint Committee on Cancer staging system. RT after treatment was defined according to the recorded data. RT0, RT1, and RT2 were defined as no RT, RT **≤**1 cm, and RT >1 cm, respectively. The records of the CA125 status before treatment were collected. CA125 results were reported as positive/elevated (Code 010) if they were >35 U/ml before treatment and those with CA125 ranges from 0 to 35 were recorded as negative/normal (Code 020). According to the previous studies, the median age of EOC at diagnosis was 50 years and more than half of all EOC cases occurred in those aged ≥65 years.^16^, ^17^ Therefore, the specific age categories were grouped as <50 years, 50–64 years, and ≥65 years. The primary endpoint of this study was cancer‐specific survival (CSS), which was defined as the data from initial treatment to the death from ovarian cancer

### 2.3 Statistical analysis

We performed a Chi‐squared test to assess comparisons between NACT and PDS groups. Multivariate logistic regression was used to determine the independent predictive indicators related to RT status. CSS was calculated using Kaplan–Meier curves and compared using the log‐rank test. A multivariate Cox proportional hazards model was conducted to determine the independent prognostic factors for CSS. Sensitivity analyses were conducted to determine the prognostic effect of RT on CSS after stratification by treatment groups. Data analyses were conducted by the SPSS version 22 (SPSS Inc.). *p*‐value below 0.05 was considered statistically significant.

## 3 RESULTS

### 3.1 Patient baseline characteristics

**FIGURE 1:**
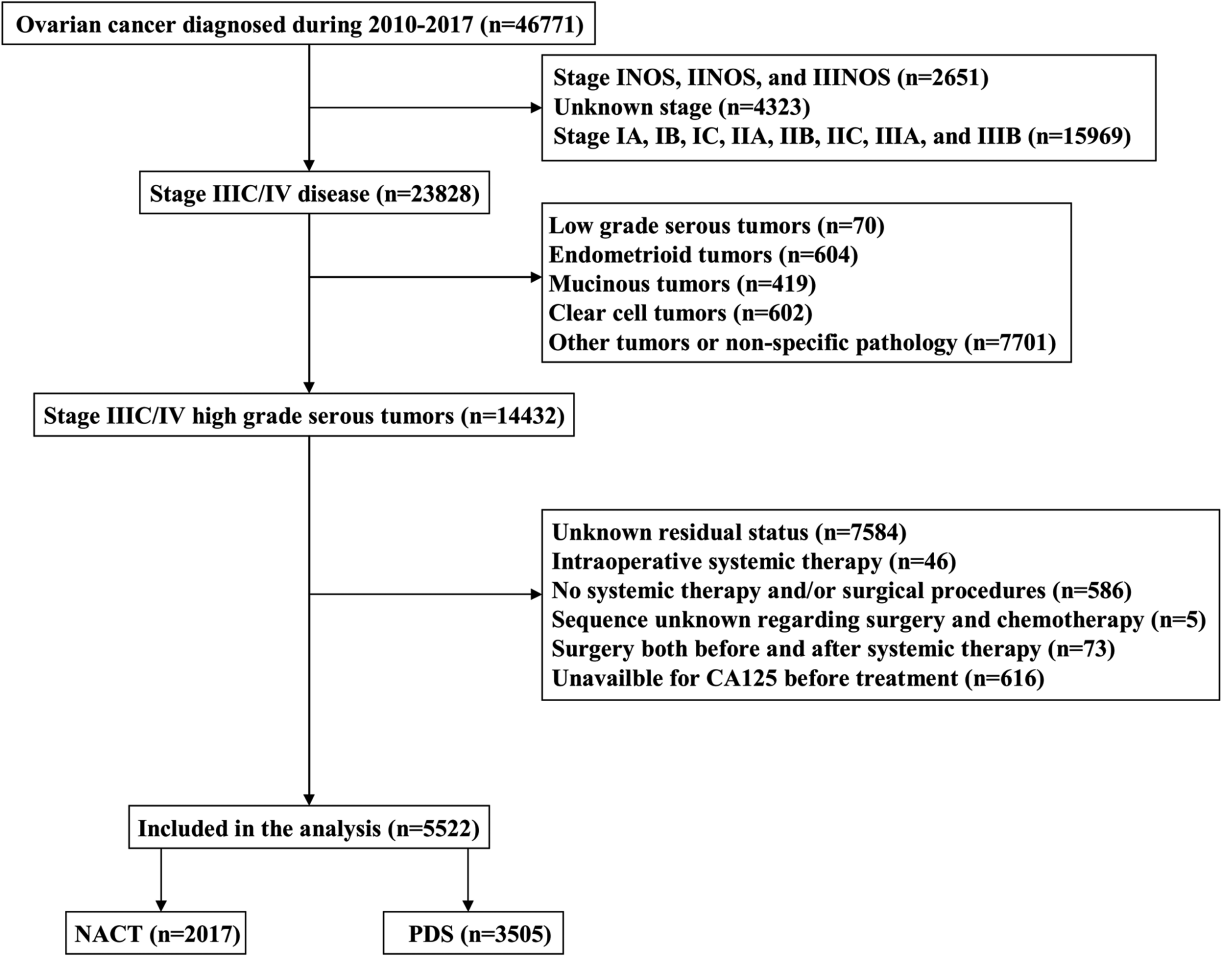
Patient selection procedure

We included 5522 patients in the analysis (Figure 1). The patient baseline characteristics are summarized in Table 1. Of these patients, 64.6% (*n* = 3565) were stage III disease, 97.2% (*n* = 5366) had CA125 >35 U/ml before treatment, and 84.1% (*n* = 4646) were a white race; 2971 (53.8%), 1637 (29.6%), and 914 (16.6%) patients had RT0, RT1, and RT2, respectively.

**TABLE 1:** Patient baseline characteristics

Variables	*n*	NACT (%)	PDS (%)	*p*
Year of diagnosis				
2010–2013	2615	797 (39.5)	1818 (51.9)	<0.001
2014–2017	2907	1220 (60.5)	1687 (48.1)	
Age at diagnosis (years)				
<50	826	199 (9.9)	627 (17.9)	<0.001
50–64	2350	828 (41.1)	1522 (43.4)	
≥65	2346	990 (49.1)	1356 (38.7)	
Race				
White	4646	1673 (82.9)	2973 (84.8)	0.176
Black	385	149 (7.4)	236 (6.7)	
Other	491	195 (9.7)	296 (8.4)	
Stage				
IIIC	3565	956 (47.4)	2609 (74.4)	<0.001
IV	1957	1061 (52.6)	896 (25.6)	
CA125 level before treatment				
≤35 U/ml	156	35 (1.7)	121 (3.5)	<0.001
>35 U/ml	5366	1982 (98.3)	3384 (96.5)	
Residual tumor				
RT0	2971	1036 (51.4)	1935 (53.8)	<0.001
RT1	1637	594 (29.4)	1043 (29.6)	
RT2	914	387 (19.2)	527 (16.6)	

Abbreviations: NACT, neoadjuvant chemotherapy; PDS, primary debulking surgery; RT0, no residual tumor; RT1, residual tumor ≤1 cm; RT2, residual tumor >1 cm.

**FIGURE 2:**
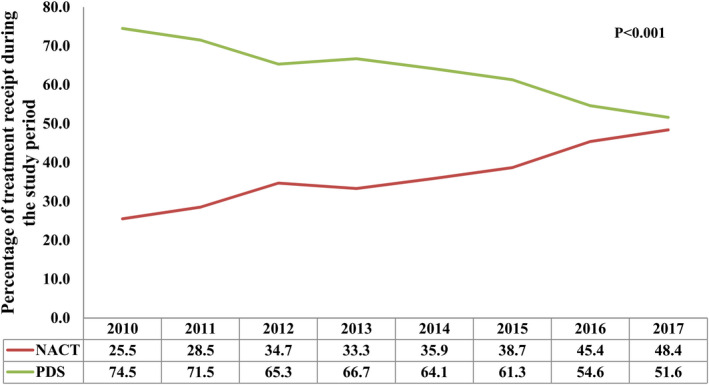
Change in use of neoadjuvant chemotherapy over time

A total of 2017 (36.5%) and 3505 (63.5%) patients received NACT and PDS, respectively. Patients diagnosis in 2014–2017, aged ≥65 years, stage IV disease, CA125 >35 U/ml, and RT2 status were more likely to receive NACT (all *p* < 0.001). The receipt of NACT was significantly increased during the study period. There was 25.5% of patients receiving NACT in 2010 and was 48.4% in 2017 (*p* < 0.001) (Figure 2).

### 3.2 Predictive factors related to residual tumors

We conducted a multivariate logistic regression analysis to determine the independent predictive factors associated with RT status. The results showed that patients diagnosed in 2010–2013, aged ≥65 years, other race, stage IV disease, and CA125 >35 U/ml before treatment were the independent predictive factors associated with RT after surgery (all *p* < 0.05). CA125 >35 U/ml had the highest chance of RT (odds ratio 3.398, *p* < 0.001) (Table 2). However, the receipt of NACT was not related to a higher chance of complete resection compared to those receiving PDS (*p* = 0.098).

**TABLE 2:** Independent predictive factors related to residual tumor (no residual tumor vs. residual tumor size ≤1 cm and >1 cm)

Variables	OR	95% CI	*p*
Year of diagnosis			
2010–2013	1		
2014–2017	0.733	0.658–0.817	<0.001
Age at diagnosis (years)			
<50	1		
50–64	1.118	0.950–1.315	0.180
≥65	1.292	1.097–1.522	0.002
Race			
White	1		
Black	1.081	0.876–1.344	0.467
Other	0.755	0.623–0.915	0.004
Stage			
IIIC	1		
IV	1.223	1.089–1.373	0.001
CA125 level before treatment			
≤35 U/ml	1		
>35 U/ml	3.398	2.290–5.041	<0.001
Treatment			
NACT	1		
PDS	0.903	0.803–1.015	0.087

Abbreviations: CI, confidence interval; NACT, neoadjuvant chemotherapy; OR, odds ratio; PDS, primary debulking surgery.

### 3.3 Survival and prognostic analyses

The median follow‐up was 34 months (range, 0–107 months). A total of 2770 patients died and 87.8% (*n* = 2433) of them died from ovarian cancer. The 3‐ and 5‐year CSS were 66.6% and 46.2%, respectively.

**FIGURE 3:**
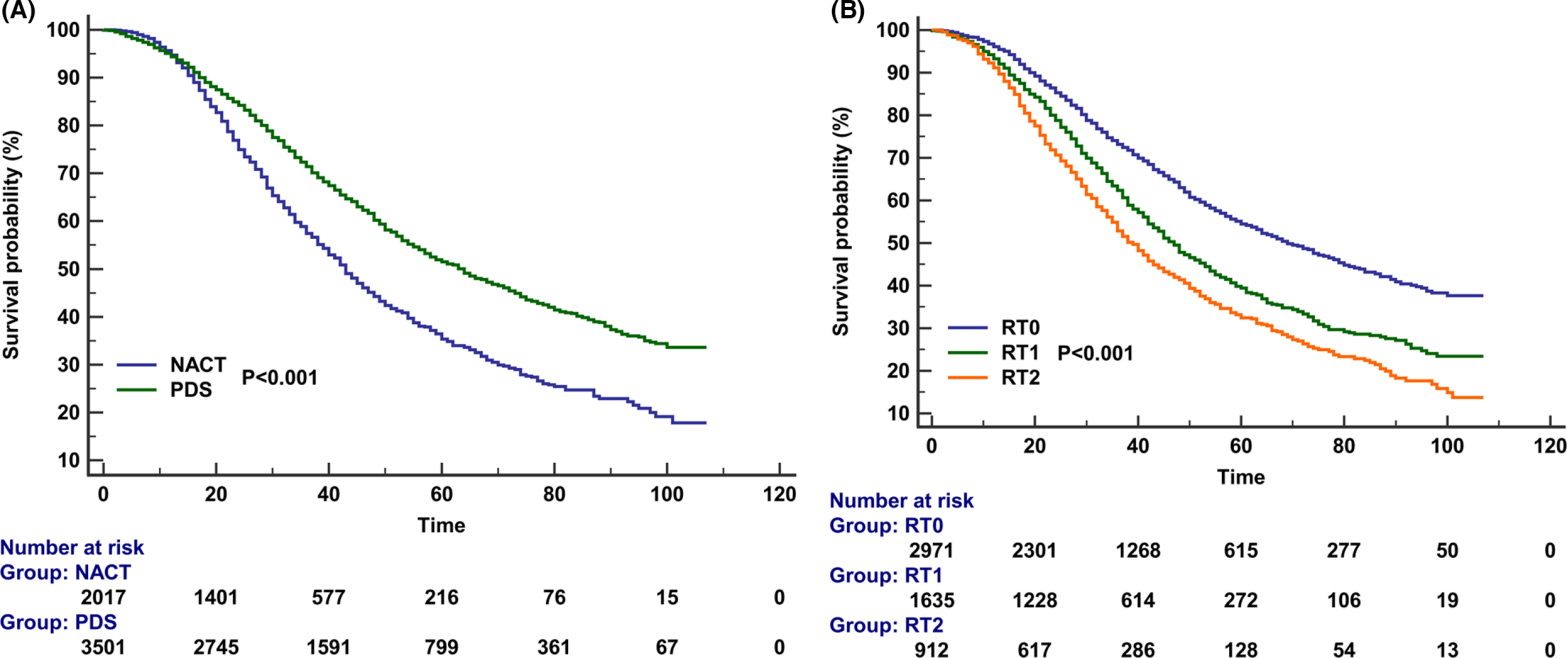
Kaplan–Meier curves for cancer‐specific survival according to the treatment receipt (A) and residual tumor status (B)

The results of multivariate Cox regression analysis indicated that patients receiving PDS had significantly better CSS than those receiving NACT (hazard ratio [HR] 0.735, *p* < 0.001). The survival curves between those receiving NACT and PDS has listed in Figure 3A. The 5‐year CSS was 35.3% and 51.1% in those receiving NACT and PDS (*p* < 0.001), respectively. In addition, patients with RT1 (HR 1.485, *p* < 0.001) and RT2 (HR 1.783, *p* < 0.001) had significantly inferior CSS compared to those who had RT0. The survival curves among the RT status have listed in Figure 3B. Moreover, age, race, tumor stage, and the levels of CA125 before treatment were also the independent prognostic factors associated with CSS (Table 3).

**TABLE 3:** Multivariate Cox regression analysis for prognostic factors associated with cancer‐specific survival in the entire cohort

Variables	HR	95% CI	*p*
Year of diagnosis			
2010–2013	1		
2014–2017	0.923	0.844–1.009	0.078
Age at diagnosis (years)			
<50	1		
50–64	1.166	1.027–1.324	0.017
≥65	1.362	1.200–1.547	<0.001
Race			
White	1		
Black	1.186	1.018–1.382	0.029
Other	0.899	0.773–1.045	0.164
Stage			
IIIC	1		
IV	1.284	1.179–1.399	<0.001
CA125 level before treatment			
≤35 U/ml	1		
>35 U/ml	1.365	1.024–1.819	0.034
Residual tumor			
RT0	1		
RT1	1.485	1.356–1.627	<0.001
RT2	1.783	1.605–1.981	<0.001
Treatment			
NACT	1		
PDS	0.735	0.673–0.802	<0.001

Abbreviations: CI, confidence interval; HR, hazard ratio; NACT, neoadjuvant chemotherapy; PDS, primary debulking surgery; RT0, no residual tumor; RT1, residual tumor ≤1 cm; RT2, residual tumor >1 cm.

Sensitivity analyses were conducted to determine the role of NACT on CSS after stratification by the years of diagnosis, age, race, tumor stage, the levels of CA125, and RT status (Table 4). The results indicated that patients receiving PDS had significantly better CSS compared to those receiving NACT regardless of the years of diagnosis, age, and the levels of CA 125. However, the CSS was comparable between NACT and PDS groups in stage IV disease, RT2, and non‐white race.

**TABLE 4:** Sensitivity analyses to determine the effect of neoadjuvant chemotherapy related to cancer‐specific survival by specific subgroups

Variables	Treatment	HR	95% CI	*p*
Year of diagnosis				
2010–2013	PDS vs. NACT	0.718	0.664–0.801	<0.001
2014–2017	PDS vs. NACT	0.761	0.659–0.879	<0.001
Age at diagnosis (years)				
<50	PDS vs. NACT	0.572	0.444–0.736	<0.001
50–64	PDS vs. NACT	0.741	0.648–0.847	<0.001
≥65	PDS vs. NACT	0.786	0.692–0.892	<0.001
Race				
White	PDS vs. NACT	0.724	0.659–0.796	<0.001
Black	PDS vs. NACT	0.804	0.576–1.121	0.198
Other	PDS vs. NACT	0.751	0.557–1.013	0.061
Stage				
IIIC	PDS vs. NACT	0.617	0.551–0.691	<0.001
IV	PDS vs. NACT	0.921	0.810–1.048	0.213
CA125 level before treatment				
≤35 U/ml	PDS vs. NACT	0.367	0.182–0.740	0.005
>35 U/ml	PDS vs. NACT	0.746	0.683–0.814	<0.001
Residual tumor				
RT0	PDS vs. NACT	0.651	0.570–0.742	<0.001
RT1	PDS vs. NACT	0.748	0.654–0.868	<0.001
RT2	PDS vs. NACT	0.931	0.776–1.119	0.447

Abbreviations: CI, confidence interval; HR, hazard ratio; NACT, neoadjuvant chemotherapy; PDS, primary debulking surgery; RT0, no residual tumor; RT1, residual tumor ≤1 cm; RT2, residual tumor >1 cm.

### 3.4 The effect of residual status on CSS after stratification by treatment groups

**FIGURE 4:**
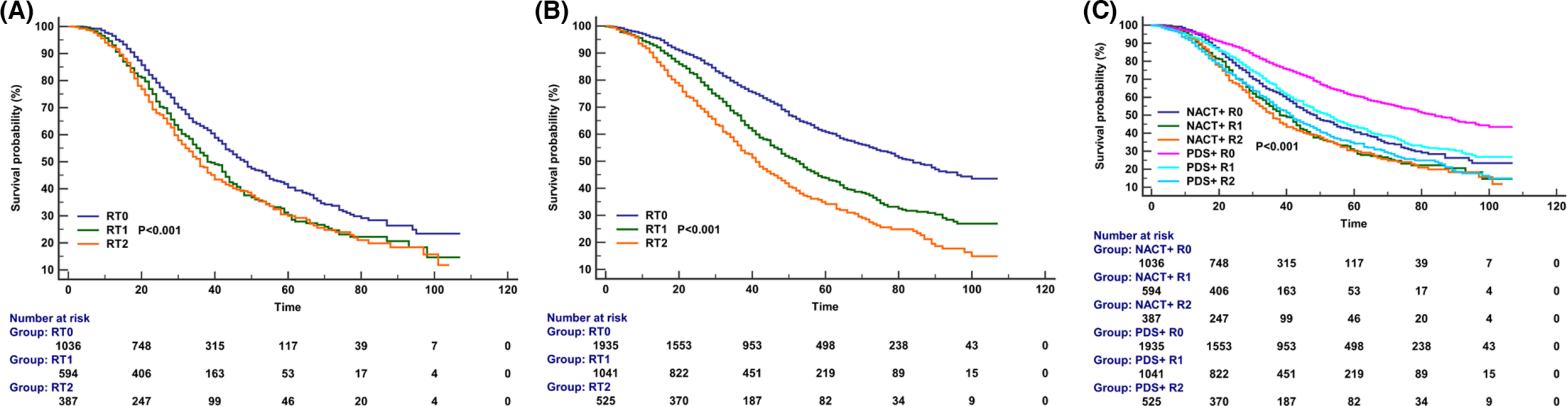
Kaplan–Meier curves for cancer‐specific survival according to the residual tumor after stratification by treatment groups (A, neoadjuvant chemotherapy group; B, primary debulking surgery group; C, entire cohort)

Finally, we conducted three multivariate Cox regression models to determine the prognostic effect of RT on CSS after stratification by treatment groups (Table 5). In the first model including patients who received NACT, the results showed that patients who had RT0 had significantly better CSS compared to those who had RT1 (*p* < 0.001), while comparable CSS was found between those who had RT1 and RT2 (*p* = 0.442). The survival curves among the RT status in the NACT group have listed in Figure 4A. In the second model including patients who received PDS, the results indicated that patients who had RT0 had a significantly better CSS compared to those who had RT1 (*p* < 0.001), and those with RT2 had a significantly inferior CSS than those who had RT1 (*p* < 0.001). The survival curves among the RT status in the NACT group have listed in Figure 4B. In the third model, including the entire cohort to investigate the CSS according to treatment received and RT status, the results indicated that compared to those with NACT + RT0, those in NACT+RT1 (HR 1.319, *p* < 0.001), NACT+RT2 (HR 1.386, *p* < 0.001), and PDS + RT2 (HR 1.340, *p* < 0.001) groups had significantly inferior CSS, and those in PDS + RT0 (HR 0.621, *p* < 0.001) had a significantly better CSS. However, similar CSS was found between those in NACT + RT0 and PDS + RT1 (HR 0.997, *p* = 0.965). The survival curves according to treatment received and RT status are listed in Figure 4C.

**TABLE 5:** Multivariate Cox regression models to determine the effect of residual tumor on cancer‐specific survival after stratification by treatment groups

Treatment	Residual tumor	HR	95% CI	*p*
NACT	RT1	1		
	RT0	0.746	0.645–0.862	<0.001
	RT2	1.070	0.900–1.273	0.442
PDS	RT1	1		
	RT0	0.630	0.561–0.708	<0.001
	RT2	1.326	1.151–1.528	<0.001
Entire cohort	NACT + RT0	1		
	NACT + RT1	1.319	1.140–1.525	<0.001
	NACT + RT2	1.386	1.177–1.630	<0.001
	PDS + RT0	0.621	0.548–0.705	<0.001
	PDS + RT1	0.997	0.875–1.136	0.965
	PDS + RT2	1.340	1.155–1.553	<0.001

Abbreviations: CI, confidence interval; HR, hazard ratio; NACT, neoadjuvant chemotherapy; PDS, primary debulking surgery; RT0, no residual tumor; RT1, residual tumor ≤1 cm; RT2, residual tumor >1 cm.

## 4 DISCUSSION

In this study, we assessed the survival outcomes between NACT and PDS in advanced EOC. In addition, we also assessed the prognostic value of the RT after NACT and PDS. Our results indicated that although the use of NACT was increased from 2010 to 2017, an inferior CSS was found in the NACT group to those in the PDS group. In addition, patients without RT had a superior CSS when treated with PDS.

Approximately 70% of EOC patients have advanced stage and optimal cytoreduction followed by adjuvant chemotherapy are the standard of care for EOC.^2^, ^3^ Before the publication of the EORTC 55971 results, the administration of NACT was less in advanced EOC. A study from Korea showed that 16.2% of patients received NACT in 2006–2010, and 48.9% of patients received NACT in 2011–2014 due to the publication of the EORTC 55971 trial.^4^ In addition, a large cohort included 36,602 stage III/IV EOC diagnosed between 2004 and 2015 from the National Cancer Database (NCDB), 27.0% (*n* = 9885) of patients treated with NACT.^18^ Moreover, the study from Meyer et al. included 1538 patients with advanced EOC, they found that the receipt of NACT was 16% between 2003 and 2010 and was 34% during 2011 and 2012 in stage IIIC disease (*p* < 0.001). There were 41% and 62% in patients with stage IV disease, respectively (*p* < 0.001).^19^ In our study, 25.5% of patients received NACT in 2010 and 48.4% in 2017 (*p* < 0.001), which was similar to the above studies. However, 68% of the respondents still believed that there was insufficient evidence to treat advanced EOC patients with NACT in the updated data of the survey from the SGO in 2016.^20^ In addition to the publication of the CHORUS and EORTC 55971 outcomes, various factors including age, tumor stage, tumor size, and co‐morbidity may also impact the choice of NACT.^21^, ^22^, ^23^, ^24^

Currently, there are still controversies regarding the use of NACT in advanced EOC. In addition to the results from randomized controlled trials,^4^, ^5^, ^6^ a meta‐analysis included 3759 patients from 4 prospective studies and 13 retrospective studies also showed that the NACT group was related to lower mortality and a better chance of complete resection than the PDS group, with no survival benefit.^25^ However, the findings from the NCDB showed that those who received NACT had a 37% higher mortality than those in the PDS group,^18^ which was also confirmed by several retrospective studies.^26^, ^27^ The difference regarding enrolled patients, surgical skills, preoperative and intraoperative evaluation may be the main reasons for the conflicting results of the above studies. The findings of our study were similar to the findings from NCDB. In the EORTC 55971 and CHORUS trials, the median OS was 24.1–30 and 22.6–29 months in those treated with NACT and PDS, respectively.^4^, ^5^ However, in our study, the median CSS time was 43 and 64 months in those receiving NACT and PDS (*p* < 0.001), respectively, which was significantly longer than the results from the above‐randomized trials. However, this approach has significant selection bias in retrospective studies because patients with poor performance status and advanced stage were more likely to treat with NACT.

Using sensitivity analysis, we found that PDS was related to a better CSS for stage IIIC patients, but not in stage IV patients. The results from recently prospective and retrospective studies confirmed our findings.^4^, ^19^, ^22^, ^23^ Thus, more studies should focus on identifying specific clinical and molecular features to help gynecological oncologists to select patients who have the greatest survival benefit from NACT. In current clinical practice, NACT is an acceptable option for selected patients, especially for those with a high tumor burden and medical comorbidities.

The reasons why those treated with NACT have impaired long‐term survival remain unclear. However, previous studies have shown that NACT might increase the risk of chemotherapy resistance due to the cancer stem cell reservoir.^28^ The study from Rauh‐Hain et al. showed that more patients developed platinum resistance in the NACT group than the PDS group (88.8% vs. 55.3%).^14^ Moreover, Lee et al. reported that 91% of patients with RT after NACT had changes in at least one of the targetable pathways, and those patients with alterations in the PI3K–AKT–mTOR signaling pathway (*p* = 0.005) and cell cycle (*p* = 0.004) had inferior OS.^29^ Therefore, the better prognostic effect of PDS may be the immediate resection of tumors that may develop chemotherapy resistance.

RT has been confirmed to be a risk factor affecting the outcomes in advanced EOC.^30^, ^31^ Although RT0 or RT1 is desirable, optimal surgery was only 25%–40% in the PDS group in the vast majority of institutions.^32^ A study from the Danish Gynecological Cancer Database (DGCD) found that patients receiving NACT had a higher chance of complete resection compared to the PDS patients (52% vs. 39%, *p* < 0.001).^23^ However, we found that whether the use of NACT did not affect the RT status of the patient, which was consistent with the results from several prospective and retrospective studies.^24^, ^33^ It should be noted that in our study, the complete resection rates of patients receiving NACT and PDS were 51.4% and 53.8%, respectively. The RT0 rate in the NACT group was similar to that of DGCD, while was significantly higher than that of DGCD in the PDS group. In addition, our study found that the levels of CA125 ≤35 U/ml had the highest chance of complete resection, but only 3.8% of patients had CA125 ≤35 U/ml. Although the SEER database did not record detailed CA125 information for EOC, a study from Japan also found that CA125 ≤30 U/ml may be a useful predictive factor for achieving complete resection.^34^

The report from the NCDB found that PDS + microscopic or no RT had the best survival, and PDS + macroscopic RT survival was similar to NACT+ microscopic or no RT, and NACT + macroscopic RT has the worst survival (*p* < 0.001).^18^ However, they did not conduct further analysis between the microscopic RT and no RT. The results in the randomized trial from Japan did not find a difference in prognosis between NACT and PDS, but they found that for patients receiving PDS, the OS in those with RT1 was significantly inferior to those with RT0, and was significantly better than those with RT2. The median OS was not estimable, 54.9, and 43.0 months in those with RT0, RT1, and RT2, respectively. However, for patients receiving NACT, the survival curves of RT1 and RT2 overlap and were significantly lower than those of patients with RT0. The median OS was 67.0, 34.0, and 32.0 months in those with RT0, RT1, and RT2, respectively.^35^ Similar results were also found from other studies.^24^, ^36^ Moreover, a long‐term study showed that the 10‐year OS was 36.0%, 10.5%, and 5.0% in those with RT0, RT1, and RT2 in the PDS cohort, respectively (*p* < 0.001), and was 10.9%, 6.2%, and 10.0% for the NACT cohort (*p* = 0.080), respectively.^37^ Our findings were similar to the above results.

In our study, the prognosis in patients with PDS + no RT was the best and was significantly better than those in NACT+ no RT group. The findings were similar to several prior studies.^21^, ^26^, ^27^ Our findings highlight that the objective of both IDS and PDS should be complete tumor resection because any RT was related to an inferior prognosis. Therefore, when complete resection does not seem feasible in the first place, it is reasonable to question the role of IDS for patients receiving NACT. In order to improve the prognosis for advanced EOC, the definition of “optimal resection” should be defined as no RT in the NACT group.

Several limitations should be acknowledged in our study. First, selection bias was unavoidable in retrospective studies. There is a lack of information regarding the reason for primary treatment selection. Second, the chemotherapy cycle, chemotherapy regimen, evaluation of chemotherapy efficacy, and the administration of maintenance treatment are also not included in the SEER program. Third, the location of the RT, the pattern of disease recurrence, and the subsequent treatment after disease recurrence are also not recorded. Finally, the SEER database classifies CA125 as positive/elevated or negative/normal without providing any information on the exact value.

In conclusion, our study suggests that PDS may be the optimal procedure for the majority of advanced EOC patients. With the increased utilization of NACT in advanced EOC, removal of all microscopic and macroscopic tumors should be the goal for those receiving NACT. More prospective studies are needed to confirm our findings.

## CONFLICT OF INTERESTS

The authors declare that there are no conflicts of interest.

## AUTHOR CONTRIBUTION

SPY and JXC drafted the manuscript. SGW acquired the datasets. JZ and SGW conceived of the study. SGW conducted the statistical analyses. JYX, JL, SGW, and JZ participated in the study design. All authors read and approved the final manuscript.

## ETHICS STATEMENT

As the SEER database consists of de‐identified information, the study was exempt from the approval process of the Institutional Review Boards of the First Affiliated Hospital of Xiamen University.

## Data Availability

Data sharing is not applicable to this article as no new data were created or analyzed in this study.
